# Impact of multimorbidity and polypharmacy on the management of patients with atrial fibrillation: insights from the BALKAN-AF survey

**DOI:** 10.1080/07853890.2020.1799241

**Published:** 2020-08-04

**Authors:** Monika Kozieł, Stefan Simovic, Nikola Pavlovic, Aleksandar Kocijancic, Vilma Paparisto, Ljilja Music, Elina Trendafilova, Anca Rodica Dan, Zumreta Kusljugic, Gheorghe-Andrei Dan, Gregory Y. H. Lip, Tatjana S. Potpara

**Affiliations:** a Liverpool Centre for Cardiovascular Science, University of Liverpool and Liverpool Heart & Chest Hospital, Liverpool, UK; b First Department of Cardiology and Angiology, Silesian Centre for Heart Diseases, Zabrze, Poland; c Cardiology Clinic, University Clinical Center of Kragujevac, Kragujevac, Serbia; d Clinical Center Sestre Milosrdnice, Zagreb, Croatia; e Cardiology Clinic, Clinical Center of Serbia, Belgrade, Serbia; f Clinic of Cardiology, University Hospital Center Mother Theresa, Tirana, Albania; g Cardiology Clinic, Medical Faculty, University Clinical Center of Montenegro, University of Podgorica, Podgorica, Montenegro; h Coronary Care Unit, National Heart Hospital, Sofia, Bulgaria; i Cardiology Department, Colentina University Hospital, Bucharest, Romania; j Cardiology Department, Clinic of Internal Medicine, Medical Faculty, University Clinical Center Tuzla, Tuzla, Bosnia and Herzegovina; k Medicine University "Carol Davila", Colentina University Hospital, Bucharest, Romania; l School of Medicine, Belgrade University, Belgrade, Serbia; m Department of Clinical Medicine, Aalborg Thrombosis Research Unit, Aalborg University, Aalborg, Denmark

**Keywords:** Atrial fibrillation, BALKAN-AF survey, multimorbidity, polypharmacy

## Abstract

**Objective:**

We investigated the impact of multimorbidity and polypharmacy on the management of atrial fibrillation (AF) patients in clinical practice and assessed factors associated with polypharmacy and oral anticoagulation (OAC) use in AF patients with multimorbidity and polypharmacy.

**Methods:**

A 14-week prospective study of consecutive non-valvular AF patients was performed in seven Balkan countries.

**Results:**

Of 2712 consecutive patients, 2263 patients (83.4%) had multimorbidity (AF + ≥2 concomitant diseases) and 1505 patients (55.5%) had polypharmacy. 1416 (52.2%) patients had both multimorbidity and polypharmacy. Overall, 1164 (82.2%) patients received OAC, 200 (14.1%) patients received antiplatelet drugs alone and 52 (3.7%) patients had no antithrombotic therapy (AT). Non-emergency centre and paroxysmal AF were significantly associated with OAC non-use in patients with multimorbidity, whilst age ≥80 years and non-emergency centre were identified to be independent predictors of OAC non-use in patients with polypharmacy.

**Conclusions:**

Multimorbidity and polypharmacy were common among AF patients in our study. AT was suboptimal and approximately 18% of multimorbid patients with polypharmacy were not anticoagulated. Pattern of AF and non-emergency centre were associated with OAC non-use in AF patients with multimorbidity, whilst non-emergency centre and age ≥80 years were associated with OAC non-use in AF patients with polypharmacy.

**Key Message**
- Multimorbidity and polypharmacy are common among patients with AF.
- Antithrombotic therapy was suboptimal in AF patients with multimorbidity and polypharmacy.
- Approximately, 18% of multimorbid patients with polypharmacy were not anticoagulated.

## Introduction

Atrial fibrillation (AF) currently affects approximately 3% of adults worldwide [1,2], and patients with AF often have multiple comorbidities [3]. Indeed, multimorbidity (i.e. the presence of two or more chronic conditions) is common among patients with AF [4–7], with hypertension, heart failure (HF), diabetes mellitus, valvular heart disease, myocardial infarction (MI), chronic obstructive pulmonary disease (COPD) and chronic kidney disease (CKD) being the most prevalent comorbidities in AF patients [8]. Up to 50% of patients with newly diagnosed AF have two or more concomitant conditions, and these patients have greater stroke and bleeding risks than non-multimorbid AF patients [9,10]. Since stroke prevention is a cornerstone of optimal AF management [11], it is critically important to provide optimal antithrombotic therapy (AT) to patients with AF and multimorbidity.

The number of concomitant diseases tends to be directly related to polypharmacy (i.e. concomitant use of five or more drugs regardless of the illness(es) they have been prescribed for and their utility [12]). Unsurprisingly, AF has been shown to be significantly correlated with polypharmacy at the time of discharge [13].

Studies assessing patients with AF and multimorbidity are scarce, and optimal management of patients with AF and multimorbidity may be challenging for physicians. The population of the Balkans (approximately 50 million inhabitants) was underrepresented in the contemporary large international AF registries [14], and data pertinent to the management of multimorbid AF patients in the Balkan region are lacking. The BALKAN-AF survey recently provided insight into contemporary management of AF patients in seven Balkan countries [15,16].

In the present post hoc analysis of the BALKAN-AF dataset, we investigated the impact of multimorbidity and polypharmacy on the management of AF patients in clinical practice and assessed factors associated with polypharmacy and oral anticoagulation (OAC) use in AF patients with multimorbidity and polypharmacy.

## Materials and methods

The BALKAN-AF survey design has been previously reported [14]. A 14-week prospective, multicentre “snapshot” registry of consecutive patients with electrocardiographically documented AF was designed by the Serbian Atrial Fibrillation Association (SAFA) and conducted from December 2014 to February 2015 in collaboration with the National Cardiology Societies and Associations or Working Groups in Albania, Bosnia & Herzegovina, Bulgaria, Croatia, Montenegro, Romania and Serbia. Patients treated by a cardiologist or an internal medicine specialist, where cardiologist was not available, were recruited by academic and non-university hospitals and outpatient health centres (a total of 49 centres). The centres were selected by the respective National Coordinator. A signed patient informed consent form was obtained before enrolment. The study protocol is concordant with the ethical guidelines of the 1975 Declaration of Helsinki.

The exclusion criteria were age <18 years and prosthetic mechanical heart valves or significant valvular disease with indications for surgical repair.

Data were collected using an electronic case report form (eCRF) designed by SAFA. The eCRF included patient characteristics, patient presentation and healthcare setting, diagnostic procedures undertaken within the last 12 months and at enrolment and AF management at enrolment and at discharge. Stroke risk was estimated using the CHA_2_DS_2_-VASc (congestive HF, hypertension, age ≥ 75 years, diabetes, stroke/transient ischaemic attack (TIA), vascular disease, age 65–74 years, sex category) score [8]. Bleeding risk was evaluated according to the HAS-BLED (hypertension, abnormal renal/liver function, stroke, bleeding history or predisposition, labile international normalized ratio (INR), elderly (>65 years), drugs or alcohol concomitantly) score [8,17]. All the cardiovascular risk factors, diseases and risk scores definitions were defined according to individual European Society of Cardiology guidelines, other guidelines, scientific statements and textbooks presented previously in supplementary information [15].

Owing to the relatively short survey period, systematic monitoring of centres and follow-up visits were not performed. National coordinators and investigators were responsible for verification of the consecutiveness of enrolled patients and correctness and completeness of entered data.

### Statistical analysis

Categorical variables were reported as absolute frequencies and percentages, and continuous variables as mean and standard deviation (SD). Comparison of categorical variables with normal distribution was calculated using Student’s *t*-test. Continuous variables with skewed distribution were compared with Mann–Whitney’s test. The descriptive analysis included baseline characteristics of patients with/without multimorbidity and with/without polypharmacy. The variables with statistically significant association on univariate logistic regression analysis were entered into multivariable logistic regression models to identify multivariable predictors of AF management. Results are expressed as odds ratio with 95% confidence interval. A two-sided *p* value of less than .05 was interpreted as statistically significant. All analyses were performed using SAS software version 9.4 (SAS Institute, Inc., Cary, NC).

## Results

**Figure 1.**
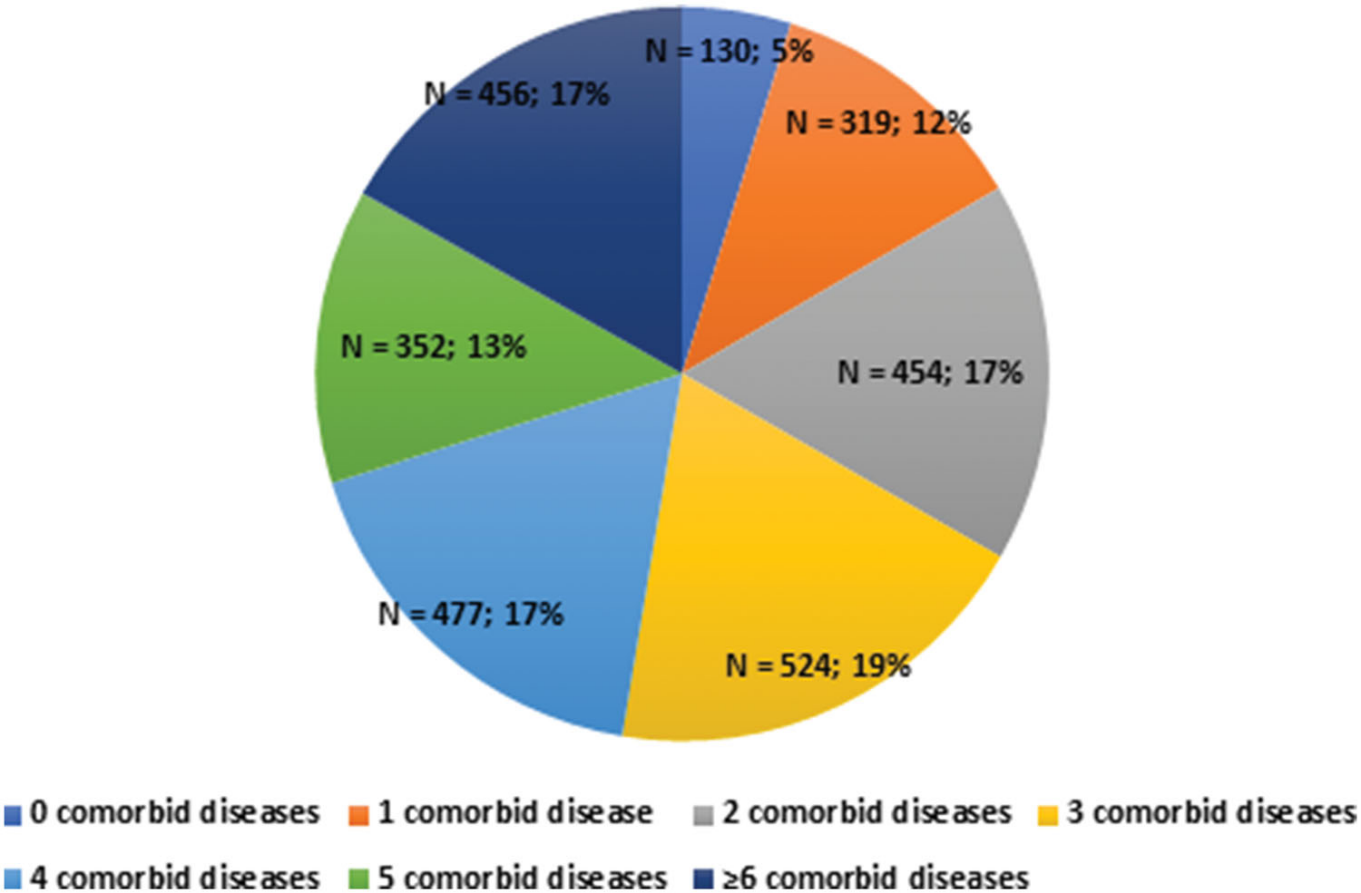
Proportion of patients with AF according to number of comorbid diseases. AF: atrial fibrillation.

Of 2712 consecutive patients, 2263 (83.4%) had multimorbidity, and polypharmacy was observed in 1505 patients (55.5%) (Supplementary Table 1 and Figure 1). Of the multimorbid patients, 1416 (62.6%) also had polypharmacy, whilst 89 (19.8%) of non-multimorbid individuals had polypharmacy.

### Demographic and AF-related characteristics

Patients with multimorbidity were older, more often female, less likely to have first-diagnosed or paroxysmal AF and more commonly had higher mean European Heart Rhythm Association (EHRA) symptom score and mean New York Heart Association (NYHA) class (all *p*<.001) compared with non-multimorbid patients. Similar findings were evident in patients with polypharmacy versus those without polypharmacy (all *p*<.001) (Supplementary Table 1).

### Physical findings and comorbidity

Patients with multimorbidity had significantly higher mean systolic blood pressure (SBP) and more comorbidities (both *p*<.05) than non-multimorbid AF patients. Similar findings were evident in patients with polypharmacy versus those without polypharmacy (all *p*<.05) (Supplementary Table 1).

### Stroke and bleeding risk factors

The mean CHA_2_DS_2_-VASc and HAS-BLED score values were higher in multimorbid patients than in those without multimorbidity (both *p*<.001) and the proportion of patients with CHA_2_DS_2_-VASc score ≥2 and HAS-BLED score ≥3 was higher in AF patients with multimorbidity than in those without multimorbidity. Similar findings were evident in patients with polypharmacy versus those without polypharmacy (all *p*<.001) (Supplementary Table 1).

### Predictors of polypharmacy

On multivariate analysis, hypertension, HF, coronary artery disease (CAD), MI, diabetes mellitus, aortic valve disease, mitral valve disease and peripheral artery disease (PAD) were independent predictors of polypharmacy in patients with AF (Table 1).

**Table 1.** Independent predictors of polypharmacy in patients with AF.

Variable	Multivariate analysis		
	OR	95% CI	*p* Value
Hypertension	3.05	2.07–4.49	<.001
HF	2.12	1.78–2.52	<.001
CAD	2.53	2.11–3.04	<.001
MI	1.56	1.13–2.15	.007
Diabetes mellitus	2.24	1.84–2.72	<.001
Aortic valve disease	1.54	1.16–2.02	.002
Mitral valve disease	1.51	1.26–1.82	<.001
PAD	1.61	1.06–2.44	.024

AF: atrial fibrillation; CAD: coronary artery disease; CI: confidence interval; HF: heart failure; MI: myocardial infarction: OR; odds ratio; PAD: peripheral artery disease.

### AF management settings

Most participants were managed in an academic healthcare facility. Patients with multimorbidity were less often treated by cardiologist and AF was less commonly the main reason for hospitalization compared to non-multimorbid AF patients (both *p*<.05). Patients with multimorbidity were more often hospitalized due to acute coronary syndromes (ACSs) and HF than those without multimorbidity (both *p*<.05). Individuals with polypharmacy were more often managed by cardiologists, and AF was less commonly the main reason for the hospitalization compared to patients without polypharmacy (both *p*<.001). ACS, HF and chronic coronary syndromes (CCSs) were also more often reasons for the hospitalization compared to those without polypharmacy (all *p*<.05) (Table 2).

**Table 2.** Atrial fibrillation management.

Variable	AF with multimorbidity *n* = 2263 (83.4%)	AF without multimorbidity *n* = 449 (16.6%)	*p* Value*	AF with polypharmacy *n* = 1505 (55.5%)	AF without polypharmacy *n* = 1207 (44.5%)	*p* Value**
AF management settings, *n* (%)						
Hospitalization due to AF	1006 (44.5)	331 (73.7)	<.001	630 (41.9)	707 (58.6)	<.001
Emergency hospitalization	1168 (51.6)	144 (32.1)	<.001	788 (52.4)	523 (43.3)	<.001
Hospitalization due to ACS	200 (8.8)	13 (2.9)	<.001	173 (11.5)	39 (3.2)	<.001
Hospitalization due to HF	652 (28.8)	12 (2.7)	<.001	464 (30.8)	199 (16.5)	<.001
Hospitalization due to CCS	107 (4.7)	24 (5.3)	.578	86 (5.7)	45 (3.7)	.017
Hospitalization due to hypertension	55 (2.4)	9 (2.0)	.587	33 (2.2)	31 (2.6)	.522
Outpatient visit	172 (7.6)	44 (9.8)	.116	124 (8.2)	92 (7.6)	.555
Academic healthcare facility	1804 (79.7)	357 (79.5)	.312	1185 (78.7)	976 (80.9)	.209
AF managed by cardiologist	1769 (78.2)	378 (84.2)	.004	1216 (80.8)	931 (77.1)	<.001
AF managed by internal medicine specialist	360 (15.9)	47 (10.5)	<.01	228 (19.1)	174 (14.4)	<.001
AF managed by GP	48 (2.1)	16 (3.6)	.073	24 (1.6)	33 (2.7)	.153
AF managed by other specialist	86 (3.8)	8 (1.8)	.057	40 (2.7)	51 (4.2)	.319
Diagnostic assessment, *n* (%)						
Routine biochemistry	1815 (80.2)	356 (79.3)	.791	1210 (80.4)	961 (79.6)	.257
Thyroid hormone measurement	761 (33.6)	182 (40.5)	<.001	530 (35.2)	412 (34.1)	.627
TTE	1817 (80.3)	330 (73.5)	<.001	1219 (81.0)	927 (76.8)	.208
Holter monitoring	585 (25.9)	123 (27.4)	.094	397 (26.4)	311 (25.8)	.580
Exercise stress test	139 (6.1)	34 (7.6)	.111	101 (6.7)	72 (6.0)	.929
Cardiac catheterization	277 (12.2)	44 (9.8)	.364	233 (15.5)	88 (7.3)	<.001
Cardiac CT scan	32 (1.4)	14 (3.1)	.004	21 (1.4)	25 (2.1)	.241
Stroke prevention, *n* (%)						
No OAC	192 (8.5)	73 (16.3)	<.001	55 (3.7)	210 (17.4)	<.001
Overall OAC	1686 (74.5)	279 (62.1)	<.001	1242 (82.5)	723 (59.9)	<.001
OAC alone	1389 (61.4)	252 (56.1)	.037	938 (62.3)	703 (58.2)	<.001
Missing	3 (0.1)	21 (4.7)	<.001	0 (0.0)	41 (3.4)	<.001
VKA	1410 (62.3)	217 (48.3)	<.001	1053 (70.0)	574 (47.6)	<.001
NOAC	276 (12.2)	62 (13.8)	.107	189 (12.6)	149 (12.3)	.865
SAPT alone	269 (11.9)	52 (11.6)	.667	141 (9.4)	180 (14.9)	<.001
ASA (alone or with OAC)	604 (26.7)	84 (18.7)	.007	447 (29.7)	241 (20.0)	<.001
DAPT alone	113 (5.0)	7 (1.6)	.003	67 (4.5)	53 (4.4)	.908
Dual antithrombotic therapy	216 (9.5)	25 (5.6)	.024	230 (15.3)	11 (0.9)	<.001
Triple antithrombotic therapy	81 (3.6)	2 (0.4)	.001	74 (4.9)	9 (0.7)	<.001
Symptom management						
AF catheter ablation	41 (1.8)	19 (4.2)	<.001	28 (1.9)	32 (2.7)	.064
ECV	69 (3.0)	28 (6.2)	.001	50 (3.3)	47 (3.9)	.168
AV node ablation with PM implantation	7 (0.3)	3 (0.7)	.130	6 (0.4)	4 (0.3)	
Rate control	1464 (64.7)	158 (35.2)	<.001	978 (65.0)	644 (53.4)	<.001
Missing	32 (1.4)	34 (7.6)	<.001	5 (0.3)	61 (5.1)	<.001
Rhythm control	685 (30.3)	215 (47.9)	<.001	482 (32.0)	418 (34.6)	.152
Digoxin	620 (27.4)	35 (7.8)	<.001	501 (33.3)	154 (12.8)	<.001
Calcium channel blockers	122 (5.4)	8 (1.8)	.001	448 (29.8)	94 (7.8)	<.001
Beta blockers	1685 (74.5)	276 (61.5)	<.001	1256 (83.5)	705 (58.4)	<.001
Propafenone	161 (7.1)	85 (18.9)	<.001	118 (7.8)	128 (10.6)	.005
Flecainide	1 (0.0)	3 (0.7)	.001	1 (0.1)	3 (0.2)	.206
Sotalol	17 (0.8)	4 (0.9)	.641	14 (0.9)	7 (0.6)	.339
Amiodarone	542 (24.0)	120 (26.7)	.024	435 (28.9)	227 (18.8)	<.001
Other therapy, *n* (%)						
ACEi	1108 (49.0)	156 (34.7)	<.001	862 (57.3)	402 (33.3)	<.001
AT1 receptor blockers	476 (21.0)	41 (9.1)	<.001	384 (25.5)	133 (11.0)	<.001
Loop diuretics	1079 (47.7)	41 (9.1)	<.001	851 (56.5)	269 (22.3)	<.001
Statins	1028 (45.4)	80 (17.8)	<.001	880 (58.5)	228 (18.9)	<.001
Polypharmacy	1416 (62.6)	89 (19.8)	<.001			
Number of drugs, mean (SD)	4.9 ± 1.5	3.4 ± 1.5	<.001	5.9 ± 0.9	4.0 ± 3.2	<.001

AF: atrial fibrillation; ACEi: angiotensin-converting enzyme inhibitors; ACS: acute coronary syndrome; ASA: acetylsalicylic acid; AV: atrioventricular; CCS: chronic coronary syndrome; CT: computed tomography; DAPT: dual antiplatelet therapy; ECV: electrical cardioversion; GP: general practitioner; HF: heart failure; NOAC: non-vitamin K oral anticoagulants; OAC: oral anticoagulation; PM: pacemaker; SAPT: single antiplatelet therapy; TTE: transthoracic echocardiography; VKA: vitamin K antagonists.

* *p* Values for patients with and without multimorbidity.

** *p* Values for patients with and without polypharmacy.

### Diagnostic assessment

Transthoracic echocardiography and cardiac computed tomography were more often performed in patients with multimorbidity compared to those without multimorbidity (both *p*<.05). Cardiac catheterization was more often performed in patients with polypharmacy compared to than in those without polypharmacy (all *p*<.05) (Table 2).

### Stroke prevention strategies

Overall, OACs alone or in combination with antiplatelet (AP) drugs were used in 1686 (74.5%) of patients with AF multimorbidity and in 279 (62.1%) of patients without multimorbidity (*p*<.001). Patients with multimorbidity were more often prescribed VKA, aspirin alone or in combination with OAC, dual AP therapy alone, dual AT and triple AT (all *p*<.05) than patients without multimorbidity. There were no significant differences in the use of NOACs among multimorbid versus non-multimorbid patients and in patients with versus those without polypharmacy. Multimorbid patients were less likely to receive no AT than those non-multimorbid (*p*<.001) (Table 2).

Overall, OAC alone or in combination with AP drugs was used in 1242 (82.5%) of patients with AF and polypharmacy and in 723 (59.9%) of those with AF without polypharmacy (*p*<.001). Patients with polypharmacy were more often prescribed VKA, aspirin alone or in combination with OAC, dual and triple AT (all *p* ≪ .001) than patients without polypharmacy, see Table 2.

### Arrhythmia-directed strategies

Rate control strategy was more frequently used in patients with multimorbidity whereas rhythm control strategy was more commonly used in patients without multimorbidity, both *p*<.001. Patients with multimorbidity were less likely to have AF catheter ablation or electric cardioversion (both *p*<.05), more likely to use digoxin, calcium channel blockers or beta-blockers (all *p*<.05) and less likely to receive amiodarone or propafenone (both *p*<.05) than those without multimorbidity. Rate control strategy, digoxin, calcium channel blockers, beta blockers, propafenone and amiodarone were more frequently used in patients with polypharmacy compared to individuals without polypharmacy (all *p*<.05), see Table 2.

### Other therapies

Multimorbid AF patients were more likely to receive angiotensin-converting enzyme inhibitors (ACEi), AT1 receptor blockers, loop diuretics and statins, and polypharmacy was more common among these patients than in patients without multimorbidity (Table 2).

Similar findings were evident in patients with polypharmacy versus those without polypharmacy (all *p*<.001) (Table 2).

### Characteristics of AF patients with both multimorbidity and polypharmacy

AF patients with both multimorbidity and polypharmacy were older than those without multimorbidity and polypharmacy (*p*<.001). They were less likely to have first-diagnosed AF and paroxysmal AF than patients without multimorbidity and polypharmacy (both *p*<.001). They had higher mean EHRA symptom score and mean NYHA class than those without multimorbidity and polypharmacy (both *p*<.001).

Patients with both multimorbidity and polypharmacy had higher mean CHA_2_DS_2_-VASc and mean HAS-BLED score than individuals without multimorbidity and polypharmacy (both *p*<.001). They were less likely to be hospitalized because of AF than those without multimorbidity and polypharmacy (*p*<.001) (Supplementary Table 2).

Patients with both multimorbidity and polypharmacy were more likely to be medicated with OAC alone or in combination with AP agents, VKA, aspirin alone or with OAC, dual and triple AT than those without multimorbidity and polypharmacy (all *p*<.001). They were also more likely to receive rate control strategy, digoxin, calcium channel blockers, amiodarone, ACEi, AT1 receptor blockers, loop diuretics and statins than patients without multimorbidity and polypharmacy (all *p*<.001) (Supplementary Table 2).

### Predictors of OAC use in patients with multimorbidity

On multivariate analysis, age, a capital city-located healthcare facility, structural heart diseases, diabetes mellitus and rate control strategy were independent predictors of OAC use, whereas non-emergency centre and paroxysmal AF were significantly associated with OAC non-use in patients with multimorbidity (Table 3).

**Table 3.** Independent predictors of OAC use in AF patients with polypharmacy or multimorbidity.

OAC use in AF patients with multimorbidity	Multivariate analysis		
	OR	95% CI	*p* Value
Age	1.16	1.08–1.24	<.001
*Capital city*	1.39	1.17–1.66	<.001
Non-emergency centre	0.52	0.41–0.65	<.001
Paroxysmal AF	0.47	0.40–0.57	<.001
Hypertension	3.49	2.84–4.29	<.001
HF	1.82	1.51–2.19	<.001
DCM	2.72	1.83–4.06	<.001
Diabetes mellitus	1.68	1.37–2.06	.001
Mitral valve disease^a^	1.98	1.64–2.41	<.001
Rate control	1.82	1.54–2.15	<.001
OAC use in AF patients with polypharmacy			
Non-emergency centre	0.68	0.56–0.83	<.001
Hypertension	3.51	2.85–4.43	<.001
Heart failure	1.86	1.56–2.21	<.001
CAD	2.73	2.25–3.31	<.001
DCM	1.97	1.40–2.77	<.001
Diabetes	2.05	1.65–2.55	<.001
Mitral valve disease^a^	1.45	1.19–1.77	.001
Age ≥ 80 years	0.65	0.52–0.81	<.001

AF: atrial fibrillation; CAD: coronary artery disease; CI: confidence interval; DCM: dilated cardiomyopathy; HF: heart failure; OAC: oral anticoagulants; OR: odds ratio.

^a^ Mild to moderate regurgitation.

### Predictors of OAC use in patients with polypharmacy

Whilst sharing the same multivariable predictors of OAC use as in multimorbid patients, age ≥80 years and non-emergency centre were identified to be independent predictors of OAC non-use, whereas structural heart disease and diabetes mellitus were independent predictors of OAC use in patients with polypharmacy (Table 3).

### Characteristics of multimorbid patients with newly-diagnosed AF or history of AF

Multimorbid patients with newly diagnosed AF had higher mean EHRA symptom score than multimorbid AF patients with history of AF (*p*<.05). They were less likely to have previous stroke/TIA, HF, prior PCI/stenting, DCM, mitral valve disease than multimorbid AF patients with history of AF (all *p*<.05). Mean CHA_2_DS_2_-VASc and HAS-BLED score values were lower in multimorbid patients with newly diagnosed AF than in multimorbid patients with history of AF (both *p*<.05) (Supplementary Table 6).

### Predictors of OAC use in multimorbid patients with newly diagnosed AF

Age, paroxysmal AF, CAD and MI were independent predictors of OAC non-use in multimorbid patients with newly diagnosed AF. Capital city, DCM, AF being the main reason for the visit and rate control strategy were independent predictors of OAC use in multimorbid patients with newly diagnosed AF (Supplementary Table 7).

## Discussion

Our study provides a novel data on “real-world” practice from the largest published prospective dataset from the Balkan region which has been largely underrepresented in prior registries and reveals several region-specific unmet needs and knowledge gaps regarding management of AF patients with multimorbidity and polypharmacy.

In this study, multimorbidity was present in 83.4% and polypharmacy was observed in 55.5% of the participants. Several studies have demonstrated that AF patients have significantly greater prevalence of concomitant diseases than age- and sex-matched controls [5–7]. In our study, an average number of comorbid conditions was lower than in a US study [6]. Our data demonstrate that the prevalence of polypharmacy among AF patients in the BALKAN-AF survey is high and consistent with other studies [18,19]. Moreover, approximately 20% of patients without multimorbidity had polypharmacy, possibly owing to simultaneous implementation of different treatment guidelines and inappropriate drug prescriptions [20].

The main findings of our study were as follows: (i) patients with both multimorbidity and polypharmacy were older, more symptomatic and more likely to have permanent AF, with higher stroke and bleeding risk in comparison to those without multimorbidity or polypharmacy, (ii) in multimorbid patients with polypharmacy AF was less often the main reason for hospitalization, but they more often received OAC (mostly a VKA), aspirin (alone or with OAC), dual and triple AT, and rate control strategy compared with patients without multimorbidity or polypharmacy and (iii) the location and type of healthcare facility, AF pattern and structural heart disease significantly influenced the stroke prevention strategies in AF patients with multimorbidity or polypharmacy.

The majority of AF multimorbid patients were prescribed OAC (74.5%). About 20% of patients with multimorbidity received SAPT only or no OAC despite high risk of stroke and such patients should optimally be anticoagulated. Optimal uptake of OAC includes offering OAC to patients with ≥1 risk factors for stroke, decision on OAC (an NOAC or a VKA with a well-managed time in therapeutic range) and patient involvement with shared decision making [8,21]. Absolute contraindications to anticoagulation are rare and complicated decisions including discontinuation of OAC should be made by multidisciplinary AF team.

The prescription of NOAC is reduced in patients with multimorbidity or polypharmacy. Interestingly, the decreased use of NOAC is also present in relatively healthier AF patients with less prevalent comorbidities in the BALKAN-AF study. The main reason for AP drug(s) use was PCI, whereas the CHA_2_DS_2_-VASc risk factors including hypertension, diabetes mellitus and HF were independent predictors of OAC use in this analysis. Mild-to-moderate mitral valve regurgitation was also an independent predictor of OAC use in multimorbid AF patients or those with polypharmacy, whereas paroxysmal AF was significantly associated with OAC non-use in multimorbid AF patients. Notably, the use of OAC should be driven by the presence of CHA_2_DS_2_-VASc stroke risk factors, and the temporal pattern of AF should not drive treatment decisions about OAC use [21]. CAD and MI were associated with decreased OAC use in multimorbid patients with newly diagnosed AF in our study. According to guidelines, OAC is indicated after elective coronary stenting for stable CAD and after an ACS in AF patients at risk of stroke [8]. Suboptimal OAC use in patients with multimorbidity appears to be unmet need in patients from BALKAN-AF survey. In one study [22], the uncertainty on how to manage stroke risk and use OAC in complex patients was one of the key knowledge gaps. The location of healthcare centre in capital city was associated with higher OAC use in patients with multimorbidity in the BALKAN-AF survey. The non-emergency centre was associated with lower OAC use in AF patients with multimorbidity or polypharmacy. In another study [23], OAC was more commonly prescribed in tertiary care centres (TCCs) which are often situated in capital cities and adhere closely to recommendations on stroke prevention in AF patients [24].

In the Balkan region, factors other than evidence-based medicine played an important role when deciding whether to choose NOACs or VKAs. Possibly, VKAs were better known to physicians than NOACs were. Moreover, NOACs were not reimbursed in the participating countries (excluding Bulgaria) during the survey period. Despite AF guidelines clearly indicate NOACs over VKAs in most AF patients, the guidelines were only modestly implemented in clinical practice in Balkan countries [16]. Some multimorbid AF patients may have contraindications to NOAC (e.g. those with severely depressed renal function).

The underuse of OAC in patients aged ≥ 80 with AF and polypharmacy may be related to the concern of drug–drug interaction, adverse outcomes or falls. However, stroke risk increases with age and the absolute benefit of OAC is clearly increased as the AF patients get older [25]. Importantly, NOACs are associated with the best safety and efficacy profiles compared to VKAs in very old patients with non-valvular AF [26,27]. Our observations emphasize the importance of reviewing drug regimens for older persons with AF taking multiple medications.

In our study, multimorbidity and polypharmacy were associated with increased likelihood of rate control strategy. Interestingly, patients with multimorbidity and polypharmacy were less likely to be treated with amiodarone despite their more symptomatic status and higher prevalence of HF than patients without multimorbidity or polypharmacy.

Evidence from other studies reveals the high prevalence of polypharmacy in AF patients (40–64%) [18,28,29]. Polypharmacy in AF patients reflects the presence of multiple cardiovascular risk factors or comorbidities. Moreover, polypharmacy and multimorbidity are associated with worse clinical outcome and characterizes high-risk AF patients with many comorbid diseases [28,30]. The link between polypharmacy and decreased adherence to medications, lower quality of life and delirium has also been reported [31].

NOACs should be preferred in AF patients with polypharmacy given their lower number of drug–drug interactions compared with VKA [32,33]. No significant difference regarding the use of NOACs in multimorbid patients versus non-multimorbid patients and in patients with polypharmacy versus those without polypharmacy may be associated with low rate of NOAC in our study, local reimbursement policies or drug availability in the market.

Patients with multimorbidity were less often hospitalized for AF and more often for other reasons than patients without multimorbidity, thus reflecting a high burden of comorbid conditions in multimorbid patients. Importantly, most comorbid diseases in patients with AF are associated with an increased risk of hospitalization, and the risk is the highest in the presence of HF and CKD [6]. In our study, management in an academic healthcare facility may result in the low incidence of first diagnosed AF or paroxysmal AF. Management decisions seem to be influenced by the type of care giver and early application of emerging treatment methods by academic healthcare centres [23].

Single disease focus should be eliminated due to high prevalence of multimorbidity and polypharmacy in AF patients. Care givers should be aware, that multimorbidity and polypharmacy in AF patients is associated with greater rates of adverse events and drug interactions in those individuals. Integrated management including patient involvement and shared decision making in multimorbid AF patients should be implemented. Moreover, integrated care of AF patients with multimorbidity should promote NOACs because of their better safety profile in those patients.

### Limitations

Our study is limited by its observational and snapshot design. Since no follow-up was planned, there was no evaluation of patient outcomes. Moreover, information about patient/prescriber treatment preferences was not available. Data on reasons for OAC non-prescription were not reported. Physicians were aware that their indications on treatment would be recorded, and registries are likely to attract highly motivated subjects and their medication at enrolment may reflect better compliance. However, the enrolment of consecutive patients limited the probability for investigators to enrol mainly patients with higher compliance. This survey was limited to the inhabitants of the Balkans, but it collected the largest AF dataset from this region, which was largely underrepresented in contemporary AF registries.

## Conclusions

Multimorbidity and polypharmacy were common among AF patients in our study. Antithrombotic therapy was suboptimal (reduced prescription of OAC and NOAC, high proportion of patients on AP alone or in combination with OAC) and approximately 18% of multimorbid patients with polypharmacy were not anticoagulated. Pattern of AF and non-emergency centre were associated with OAC non-use in AF patients with multimorbidity, whilst age ≥80 years and non-emergency centre were associated with OAC non-use in AF patients with polypharmacy.

## Supplementary Material

Supplemental Material
