# Supplementary material for: Impact of multimorbidity and polypharmacy on the management of patients with atrial fibrillation: insights from the BALKAN-AF survey
Source: Ann Med. 2020 Aug 4;53(1):17–25. doi: 10.1080/07853890.2020.1799241 (PMC7877933; doi:10.1080/07853890.2020.1799241)
Supplement: Supplemental Material [file IANN_A_1799241_SM8835.docx]

**APPENDIX**

**SUPPLEMENTARY MATERIAL**

**Impact of multimorbidity and polypharmacy on the management of patients with atrial fibrillation: Insights from the BALKAN-AF survey**

Monika Kozieł, Stefan Simovic, Nikola Pavlovic_,_ Aleksandar Kocijancic, Vilma Paparisto, Ljilja Music, Elina Trendafilova, Anca Rodica Dan, Zumreta Kusljugic, Gheorghe-Andrei Dan, Gregory Y.H. Lip, Tatjana S. Potpara, on behalf of the BALKAN-AF Investigators.

Supplementary Table 1. Demographic, AF-related characteristics, physical finding, comorbidities, stroke and bleeding risk factors and baseline stroke and bleeding risk profile

| **Variable** | **AF with multimorbidity**  **n=2263 (83.4%)** | **AF without multimorbidity**  **n=449 (16.6%)** | **p-value*** | **AF with polypharmacy**  **n=1505 (55.5%)** | **AF without polypharmacy**  **n=1207 (44.5%)** | **p-value**** |
| --- | --- | --- | --- | --- | --- | --- |
| **Age, mean (SD), yrs** | 70.4±9.9 | 62.3±13.3 | <0.001 | 69.9±9.6 | 68.1±12.3 | <0.001 |
| **Age ≥75 yrs, n (%)** | 875 (38.7) | 72 (16.0) | <0.001 | 541 (35.9) | 406 (33.6) | 0.210 |
| **BMI, mean (SD), kg/m²** | 27.8±4.6 | 27.7±4.6 | 0.608 | 28.3±4.5 | 27.1±4.2 | 0.001 |
| **Male sex, n (%)** | 1217 (53.8) | 268 (59.7) | <0.001 | 805 (53.5) | 680 (56.3) | <0.001 |
| **Current smoker, n (%)** | 262 (11.6) | 78 (17.4) | <0.001 | 180 (12.0) | 169 (14.0) | 0.311 |
| **Alcohol abuse, n (%)** | 92 (4.1) | 18 (4.0) | 0.956 | 59 (3.9) | 51 (4.2) | 0.693 |
| **First-diagnosed AF, n (%)** | 496 (21.9) | 136 (30.3) | <0.001 | 315 (20.9) | 317 (26.3) | <0.001 |
| **Paroxysmal AF, n (%)** | 752 (33.2) | 208 (46.3) | <0.001 | 503 (33.4) | 457 (37.9) | <0.001 |
| **Persistent AF, n (%)** | 315 (13.9) | 68 (15.1) | 0.118 | 183 (12.2) | 136 (11.3) | 0.474 |
| **Permanent AF, n (%)** | 997 (44.1) | 136 (30.3) | <0.001 | 658 (43.7) | 430 (35.6) | <0.001 |
| **EHRA symptom score, mean (SD)** | 2.2±0.8 | 1.8±0.8 | <0.001 | 2.2±0.8 | 2.0±0.8 | <0.001 |
| EHRA I, n (%) | 409 (18.1) | 162 (36.1) | <0.001 | 279 (18.5) | 292 (24.2) | <0.001 |
| EHRA II, n (%) | 1084 (47.9) | 170 (37.9) | <0.001 | 677 (45.0) | 577 (47.8) | 0.013 |
| EHRA III, n (%) | 644 (28.5) | 68 (15.1) | <0.001 | 454 (30.2) | 258 (21.4) | <0.001 |
| EHRA IV, n (%) | 125 (5.5) | 15 (3.3) | 0.108 | 94 (6.2) | 46 (3.8) | <0.001 |
| Missing | 1 (0.0) | 24 (5.3) | <0.001 | 1 (0.1) | 34 (2.8) | 0.004 |
| **NYHA class, mean (SD)** | 1.9±1.0 | 1.1±0.5 | <0.001 | 1.9±1.1 | 1.6±0.9 | <0.001 |
| NYHA I, n (%) | 1185 (52.4) | 384 (85.5) | <0.001 | 756 (50.2) | 813 (67.4) | <0.001 |
| NYHA II, n (%) | 362 (16.0) | 16 (3.6) | <0.001 | 247 (16.4) | 131 (10.9) | <0.001 |
| NYHA III, n (%) | 525 (23.2) | 7 (1.6) | <0.001 | 365 (24.3) | 167 (13.8) | <0.001 |
| NYHA IV, n (%) | 191 (8.4) | 7 (1.6) | <0.001 | 137 (9.1) | 61 (5.1) | <0.001 |
| Missing | 0 (0.0) | 25 (5.6) | <0.001 | 0 (0.0) | 35 (2.9) | 0.003 |
| **SBP, mean (SD), mmHg** | 135.7±22.6 | 128.8±17.4 | <0.001 | 138.3±22.8 | 129.8±22.0 | <0.001 |
| **DBP, mean (SD), mmHg** | 81.2±12.5 | 80.1±10.4 | 0.089 | 82.7±12.5 | 78.9±11.5 | <0.001 |
| **Heart rate, mean (SD), beats per minute** | 90.9±27.8 | 92.4±31.5 | 0.337 | 91.6±27.2 | 90.7±29.8 | 0.416 |
| **Medical history, n (%)** |  |  |  |  |  |  |
| Hypertension | 1916 (84.7) | 205 (45.7) | <0.001 | 1322 (87.8) | 799 (66.2) | <0.001 |
| Hypertension well controlled^a^ | 1278 (56.5) | 156 (34.7) | <0.001 | 832 (55.3) | 602 (49.9) | <0.001 |
| Previous stroke/TIA | 360 (15.9) | 4 (0.9) | <0.001 | 221 (14.7) | 143 (11.8) | <0.001 |
| CAD | 814 (36.0) | 7 (1.6) | <0.001 | 594 (39.5) | 227 (18.8) | <0.001 |
| MI | 365 (16.1) | 4 (0.9) | <0.001 | 281 (18.7) | 88 (7.3) | <0.001 |
| Prior PCI/stenting | 221 (9.8) | 4 (0.9) | <0.001 | 180 (12.0) | 45 (3.7) | <0.001 |
| HF | 1147 (50.7) | 16 (3.6) | <0.001 | 791 (52.6) | 372 (30.8) | <0.001 |
| Missing | 1 (0.0) | 23 (5.1) | <0.001 | 0 (0.0) | 37 (3.1) | 0.001 |
| LVEF < 40% | 506 (22.4) | 28 (6.2) | <0.001 | 379 (25.2) | 155 (12.8) | <0.001 |
| Diabetes mellitus | 664 (29.3) | 4 (0.9) | <0.001 | 479 (31.8) | 189 (15.7) | <0.001 |
| Prior bleeding | 129 (5.7) | 4 (0.9) | <0.001 | 62 (4.1) | 71 (5.9) | 0.021 |
| Missing | 3 (0.1) | 27 (6.0) | <0.001 | 1 (0.1) | 39 (3.2) | <0.001 |
| CKD | 409 (18.1) | 2 (0.4) | <0.001 | 269 (17.9) | 142 (11.8) | <0.001 |
| Aortic valve disease | 299 (13.2) | 1 (0.2) | <0.001 | 204 (13.6) | 96 (8.0) | <0.001 |
| Mitral valve disease (mild to moderate regurgitation) | 824 (36.4) | 21 (4.7) | <0.001 | 546 (36.3) | 299 (24.8) | <0.001 |
| DCM | 215 (9.5) | 1 (0.2) | <0.001 | 162 (10.8) | 54 (4.5) | <0.001 |
| Hyperthyroidism | 115 (5.1) | 5 (1.1) | 0.465 | 67 (4.5) | 53 (4.4) | 0.277 |
| COPD | 337 (14.9) | 5 (1.1) | <0.001 | 203 (13.5) | 139 (11.5) | 0.217 |
| Sleep apnoea | 52 (2.3) | 1 (0.2) | 0.006 | 40 (2.7) | 13 (1.1) | 0.004 |
| PAD | 121 (5.3) | 1 (0.2) | <0.001 | 86 (5.7) | 36 (3.0) | 0.001 |
| Liver disease | 94 (4.2) | 2 (0.4) | <0.001 | 47 (3.1) | 49 (4.1) | 0.138 |
| Dementia | 70 (3.1) | 1 (0.2) | 0.001 | 39 (2.6) | 32 (2.7) | 0.531 |
| Hypercholesterolemia | 992 (43.8) | 28 (6.2) | <0.001 | 723 (48.0) | 297 (24.6) | <0.001 |
| Anaemia | 369 (16.3) | 4 (0.9) | <0.001 | 210 (14.0) | 163 (13.5) | 0.437 |
| Malignancy | 118 (5.2) | 1 (0.2) | <0.001 | 65 (4.3) | 54 (4.5) | 0.373 |
| Obesity | 565 (25.0) | 106 (23.6) | 0.541 | 381 (25.2) | 290 (24.0) | 0.365 |
| Number of comorbidities, mean (SD) | 4.1±1.7 | 0.7±0.5 | <0.001 | 4.8±2.3 | 3.2±2.4 | <0.001 |
| **CHA_2_DS_2_-VASc, mean (SD)** | 3.7±1.7 | 1.4±1.3 | <0.001 | 3.8±1.7 | 2.8±1.9 | <0.001 |
| **CHA_2_DS_2_-VASc=0, n (%)** | 9 (0.4) | 112 (24.9) | <0.001 | 5 (0.3) | 116 (9.6) | <0.001 |
| **CHA_2_DS_2_-VASc=1, n (%)** | 168 (7.4) | 121 (26.9) | <0.001 | 98 (6.5) | 191 (15.8) | <0.001 |
| **CHA_2_DS_2_-VASc ≥ 2, n (%)** | 2086 (92.2) | 216 (48.1) | <0.001 | 1402 (93.2) | 900 (74.6) | <0.001 |
| **HAS-BLED, mean (SD)** | 2.1±1.2 | 1.0±0.9 | <0.001 | 2.1±1.2 | 1.7±1.2 | <0.001 |
| **HAS-BLED ≥3, n (%)** | 793 (35.0) | 30 (6.7) | <0.001 | 518 (34.4) | 305 (25.3) | <0.001 |

^a^ an average systolic blood pressure ≥140 mmHg or an average diastolic blood pressure ≥90 mmHg, among patients with hypertension.

* P-values for patients with and without multimorbidity.

** P-values for patients with and without polypharmacy.

AF, atrial fibrillation; BMI, body mass index; CAD, coronary artery disease; CHA2DS2-VASc, congestive heart failure, hypertension, age ≥ 75 years, diabetes, stroke/transient ischemic attack (TIA), vascular disease, age 65 to 74 years, sex category; CKD, chronic kidney disease; COPD, chronic obstructive pulmonary disease; DBP, diastolic blood pressure; DCM, dilated cardiomyopathy; HCM, hypertrophic cardiomyopathy; EHRA, European Heart Rhythm Association; HAS-BLED: hypertension, abnormal renal/liver function, stroke, bleeding history or predisposition, labile International Normalised Ratio, elderly (age > 65 years), drugs or alcohol concomitantly; LVEF, left ventricular ejection fraction; MI, myocardial infarction; NYHA, New York Heart Association PCI, percutaneous coronary intervention; PAD, peripheral artery disease SBP, systolic blood pressure; SD, standard deviation.

Supplementary Table 2. Demographic, AF-related characteristics, physical finding, comorbidities, stroke and bleeding risk factors, baseline stroke and bleeding risk profile and AF management

| Variable | AF with both multimorbidity and polypharmacy  n=1416 (52.2%) | AF without multimorbidity and polypharmacy  n=1296 (47.8%) | p-value |
| --- | --- | --- | --- |
| Age, mean (SD), yrs | 70.2±9.4 | 67.9±12.2 | <0.001 |
| Age ≥75 yrs, n (%) | 522 (36.9) | 425 (32.8) | 0.026 |
| BMI, mean (SD), kg/m² | 28.3±4.5 | 27.1±4.1 | <0.001 |
| Male sex, n (%) | 754 (53.2) | 731 (53.1) | 0.957 |
| Current smoker, n (%) | 167 (11.8) | 173 (13.3) | 0.222 |
| Alcohol abuse, n (%) | 58 (4.1) | 52 (4.0) | 0.912 |
| First-diagnosed AF, n (%) | 289 (20.4) | 498 (38.4) | <0.001 |
| Paroxysmal AF, n (%) | 462 (32.6) | 498 (38.4) | <0.001 |
| Persistent AF, n (%) | 169 (11.9) | 150 (11.6) | 0.771 |
| Permanent AF, n (%) | 639 (45.1) | 449 (34.6) | <0.001 |
| EHRA symptom score, mean (SD) | 2.7±0.8 | 2.0±0.8 | <0.001 |
| EHRA I, n (%) | 240 (16.9) | 331 (25.5) | <0.001 |
| EHRA II, n (%) | 646 (45.6) | 608 (46.9) | 0.192 |
| EHRA III, n (%) | 437 (30.9) | 275 (21.2) | <0.001 |
| EHRA IV, n (%) | 92 (6.5) | 48 (3.7) | 0.002 |
| Missing | 1 (0.1) | 34 (2.6) | <0.001 |
| NYHA class, mean (SD) | 2.0±1.1 | 1.5±0.9 | <0.001 |
| NYHA I, n (%) | 677 (47.8) | 892 (68.8) | <0.001 |
| NYHA II, n (%) | 243 (17.2) | 135 (10.4) | <0.001 |
| NYHA III, n (%) | 364 (25.7) | 168 (13.0) | <0.001 |
| NYHA IV, n (%) | 132 (9.3) | 66 (5.1) | <0.001 |
| Missing | 0 (0.0) | 35 (2.7) | <0.001 |
| SBP, mean (SD), mmHg | 138.6±23.0 | 130.2±19.9 | <0.001 |
| DBP, mean (SD), mmHg | 82.7±12.6 | 79.1±11.6 | <0.001 |
| Heart rate, mean (SD), beats per minute | 91.6±27.0 | 90.7±29.8 | 0.379 |
| Medical history, n (%) |  |  |  |
| Hypertension | 1255 (88.6) | 866 (66.8) | <0.001 |
| Hypertension well controlled^a^ | 785 (55.4) | 649 (50.1) | <0.001 |
| Previous stroke/TIA | 221 (15.6) | 142 (11.0) | <0.001 |
| CAD | 592 (41.8) | 229 (17.7) | <0.001 |
| MI | 290 (19.8) | 89 (6.9) | <0.001 |
| Prior PCI/stenting | 179 (12.6) | 46 (3.5) | <0.001 |
| HF | 785 (55.4) | 378 (29.2) | <0.001 |
| Missing | 0 (0.0) | 37 (2.9) | <0.001 |
| LVEF < 40% | 364 (25.4) | 170 (13.1) | <0.001 |
| Diabetes mellitus | 478 (33.8) | 189 (14.6) | <0.001 |
| Prior bleeding | 62 (4.4) | 71 (5.5) | 0.133 |
| CKD | 268 (18.9) | 143 (11.0) | <0.001 |
| Aortic valve disease | 204 (14.4) | 96 (7.4) | <0.001 |
| Mitral valve disease | 542 (38.3) | 303 (23.4) | <0.001 |
| DCM | 162 (11.4) | 54 (4.2) | <0.001 |
| HCM | 31 (2.2) | 22 (1.7) | 0.356 |
| RCM | 2 (0.1) | 2 (0.2) | 0.929 |
| Hyperthyroidism | 66 (4.7) | 54 (4.2) | 0.471 |
| COPD | 203 (14.3) | 139 (10.7) | 0.011 |
| Sleep apnoea | 40 (2.8) | 13 (1.0) | 0.001 |
| PAD | 86 (6.1) | 36 (2.8) | <0.001 |
| Liver disease | 47 (3.3) | 49 (3.8) | 0.420 |
| Dementia | 39 (2.8) | 32 (2.5) | 0.734 |
| Hypercholesterolemia | 721 (50.9) | 299 (23.1) | <0.001 |
| Anaemia | 210 (14.8) | 163 (12.6) | 0.159 |
| Malignancy | 65 (4.6) | 54 (4.2) | 0.713 |
| Obesity | 358 (25.3) | 313 (24.2) | 0.382 |
| Number of comorbidities, mean (SD) |  |  |  |
| CHA_2_DS_2_-VASc, mean (SD) | 3.9±1.7 | 2.7±1.9 | <0.001 |
| CHA_2_DS_2_-VASc=0, n (%) | 3 (0.2) | 118 (9.1) | <0.001 |
| CHA_2_DS_2_-VASc=1, n (%) | 76 (5.4) | 213 (16.4) | <0.001 |
| CHA_2_DS_2_-VASc ≥ 2, n (%) | 1337 (94.4) | 965 (74.5) | <0.001 |
| HAS-BLED, mean (SD) | 2.2±1.2 | 1.7±1.3 | <0.001 |
| HAS-BLED ≥3, n (%) | 507 (35.8) | 316 (24.4) | <0.001 |
| *AF management settings, n (%)* |  |  |  |
| AF was the main reason for the hospitalization (at baseline visit) | 570 (40.3) | 767 (59.2) | <0.001 |
| Outpatient visit | 112 (7.9) | 104 (8.0) | 0.912 |
| Academic healthcare facility | 1116 (78.8) | 1045 (80.6) | 0.127 |
| AF managed by a cardiologist | 1137 (80.3) | 1010 (77.9) | 0.130 |
| *Stroke prevention (at baseline visit), n (%)* |  |  |  |
| No antithrombotic therapy | 52 (3.7) | 213 (16.4) | <0.001 |
| Overall OAC | 1164 (82.2) | 801 (61.8) | <0.001 |
| OAC alone | 881 (62.2) | 760 (58.6) | 0.057 |
| Missing | 0 (0.0) | 41 (3.2) | <0.001 |
| VKA | 991 (70.0) | 636 (49.1) | <0.001 |
| NOAC | 173 (12.2) | 165 (12.7) | 0.471 |
| SAPT alone | 134 (9.5) | 187 (14.4) | <0.001 |
| ASA (alone or with OAC) | 418 (29.5) | 270 (20.8) | <0.001 |
| DAPT alone | 66 (4.7) | 54 (4.2) | 0.656 |
| Dual antithrombotic therapy | 211 (14.9) | 30 (2.3) | <0.001 |
| Triple antithrombotic therapy | 72 (5.1) | 11 (0.8) | <0.001 |
| *Symptom management* |  |  |  |
| *Non-pharmacological AF therapies (at baseline or in the future), n (%)* |  |  |  |
| AF catheter ablation | 25 (1.8) | 35 (2.7) | 0.032 |
| ECV | 42 (3.0) | 55 (4.2) | 0.016 |
| AV node ablation with PM implantation | 5 (0.4) | 5 (0.4) | 0.979 |
| *Pharmacological AF therapies (at baseline visit), n (%)* |  |  |  |
| Rate control | 939 (66.3) | 683 (52.7) | <0.001 |
| Missing | 5 (0.4) | 67 (5.2) | <0.001 |
| Rhythm control | 435 (30.7) | 465 (35.9) | 0.004 |
| Digoxin | 482 (34.0) | 173 (13.3) | <0.001 |
| Calcium channel blockers | 421 (29.7) | 121 (9.3) | <0.001 |
| Beta blockers | 1183 (83.5) | 778 (60.0) | <0.001 |
| Propafenone | 98 (6.9) | 148 (11.4) | <0.001 |
| Flecainide | 1 (0.1) | 3 (0.2) | 0.261 |
| Sotalol | 12 (0.8) | 9 (0.7) | 0.704 |
| Amiodarone | 400 (28.2) | 262 (20.2) | <0.001 |
| *Other therapy (at baseline), n (%)* |  |  |  |
| ACEi | 811 (57.3) | 453 (35.0) | <0.001 |
| AT1 receptor blockers | 365 (25.8) | 152 (11.7) | <0.001 |
| Loop diuretics | 829 (58.5) | 291 (22.5) | <0.001 |
| Statins | 836 (59.0) | 272 (21.0) | <0.001 |
| Number of drugs, mean (SD) | 5.9±0.9 | 3.4±1.1 | <0.001 |

^a^an average systolic blood pressure <140 mmHg or an average diastolic blood pressure <90 mmHg, among patients with hypertension.

AF, atrial fibrillation; ASA, acetylsalicylic acid; BMI, body mass index; CAD, coronary artery disease, CHA_2_DS_2_-VASc, congestive heart failure, hypertension, age ≥ 75 years, diabetes, stroke/transient ischemic attack (TIA), vascular disease, age 65 to 74 years, sex category; CKD, chronic kidney disease; COPD, chronic obstructive pulmonary disease; DAPT, dual antiplatelet therapy; DBP, diastolic blood pressure; DCM, dilated cardiomyopathy; EHRA, European Heart Rhythm Association; HAS-BLED: hypertension, abnormal renal/liver function, stroke, bleeding history or predisposition, labile International Normalised Ratio, elderly (age > 65 years), drugs or alcohol concomitantly; HCM, hypertrophic cardyomyopathy; HF, heart failure; LVEF, left ventricular ejection fraction; MI, myocardial infarction; NOAC, non-vitamin K oral anticoagulant; NYHA, New York Heart Association; OAC, oral anticoagulation; PAD, peripheral artery disease; PCI, percutaneous coronary intervention; SAPT, single antiplatelet therapy; SBP, systolic blood pressure; SD, standard deviation; TIA, transient ischaemic attack; VKA, vitamin K antagonists.

Supplementary Table 3. Independent predictors of polypharmacy presence in patients with AF

|  | **Univariate analysis** | | |
| --- | --- | --- | --- |
| **Variable** | **OR** | **95% Confidence interval** | **p-value** |
| Female sex | 1.18 | 1.01-1.38 | 0.030 |
| Current smoker | 0.92 | 0.63-1.35 | 0.689 |
| Hypertension | 3.36 | 2.76-4.09 | <0.001 |
| Heart failure | 2.37 | 2.02-2.78 | <0.001 |
| Coronary artery disease | 2.71 | 2.26-3.24 | <0.001 |
| Myocardial infarction | 1.42 | 1.04-1.94 | 0.027 |
| Diabetes mellitus | 2.41 | 2.00-2.92 | <0.001 |
| Aortic valve disease | 1.81 | 1.40-2.34 | <0.001 |
| Mitrale valve disease | 1.72 | 1.46-2.04 | <0.001 |
| Previous stroke | 1.25 | 0.97-1.61 | 0.080 |
| Peripheral artery disease | 1.90 | 1.28-2.83 | 0.001 |
| CKD | 1.63 | 1.31-2.03 | <0.001 |
| Hepatic disease | 0.73 | 0.48-1.10 | 0.139 |
| COPD | 1.15 | 0.91-1.45 | 0.218 |

AF, atrial fibrillation; CI, confidence interval; CKD, chronic kidney disease; COPD, chronic obstructive pulmonary disease; OR, odds ratio.

Supplementary Table 4. Independent predictors of OAC use in patients with AF and multimorbidity

|  | **Univariate analysis** | |  |
| --- | --- | --- | --- |
| **Variable** | **OR** | **95% Confidence interval** | **p-value** |
| Mean age, years | 1.02 | 1.01-1.03 | <0.001 |
| Male sex | 0.98 | 0.96-0.99 | <0.001 |
| Capital city | 1.24 | 1.06-1.45 | 0.006 |
| Non-emergency centre | 0.59 | 0.48-0.74 | <0.001 |
| University centre | 1.40 | 1.09-1.79 | <0.001 |
| Cardiologist | 0.90 | 0.73-1.11 | 0.137 |
| Alcohol abuse | 0.84 | 0.54-1.30 | 0.781 |
| Paroxysmal AF | 0.47 | 0.39-0.56 | <0.001 |
| Obesity | 1.02 | 0.84-1.25 | 0.324 |
| Hypertension | 3.41 | 2.76-4.22 | <0.001 |
| Heart failure | 2.05 | 1.70-2.48 | <0.001 |
| CAD | 1.47 | 1.24-1.76 | <0.001 |
| MI | 0.84 | 0.62-1.13 | 0.248 |
| PAD | 1.09 | 0.59-1.99 | 0.004 |
| DCM | 2.80 | 1.91-4.10 | <0.001 |
| Diabetes | 1.77 | 1.45-2.15 | <0.001 |
| Mitral valve disease | 2.15 | 1.77-2.61 | <0.001 |
| Aortic valve disease | 1.10 | 0.83-1.46 | 0.497 |
| CKD | 1.89 | 1.49-2.42 | <0.001 |
| Anemia | 0.94 | 0.74-1.19 | 0.445 |
| Malignancy | 1.21 | 0.81-1.80 | 0.254 |
| Prior stroke | 0.81 | 0.59-1.10 | 0.659 |
| Prior TIA | 1.13 | 0.70-1.82 | 0.359 |
| Age 65-74 | 1.27 | 1.04-1.55 | 0.037 |
| Age≥75 | 1.16 | 0.99-1.37 | 0.064 |
| Hypertension not well controlled | 1.16 | 0.95-1.42 | 0.165 |
| Liver disease | 0.85 | 0.51-1.42 | 0.766 |
| Bleeding events | 1.30 | 0.83-2.04 | 0.135 |
| AF was the main reason for the visit | 1.13 | 0.93-1.37 | 0.097 |
| Outpatient visit | 0.67 | 0.48-0.92 | 0.043 |
| Rate control | 1.81 | 1.41-2.32 | <0.001 |
| Rhythm control | 0.99 | 0.76-1.28 | 0.641 |

AF, atrial fibrillation; CAD, coronary artery disease; CI, confidence interval; CKD, chronic kidney disease; DCM, dilated cardiomyopathy; MI, myocardial infarction; OAC, oral anticoagulants; OR, odds ratio; PAD, peripheral artery disease; TIA, transient ischaemic attack.

Supplementary Table 5. Independent predictors of OAC use in patients with AF and polypharmacy

|  | **Univariate analysis** | | |
| --- | --- | --- | --- |
| **Variable** | **OR** | **95% Confidence interval** | **p-value** |
| Mean age, years | 1.01 | 0.99-1.02 | 0.075 |
| Male sex | 0.97 | 0.98-0.99 | <0.001 |
| Capital city | 1.32 | 1.13-1.53 | <0.001 |
| Non-emergency centre | 0.81 | 0.66-0.99 | 0.042 |
| University centre | 0.88 | 0.69-1.13 | 0.327 |
| Cardiologist | 1.27 | 1.03-1.57 | 0.022 |
| Alcohol abuse | 0.87 | 0.59-1.29 | 0.510 |
| Paroxysmal AF | 0.59 | 0.50-0.70 | <0.001 |
| Obesity | 0.95 | 0.79-1.14 | 0.605 |
| Hypertension | 3.12 | 2.50-3.89 | <0.001 |
| Heart failure | 1.84 | 1.56-2.18 | <0.001 |
| CAD | 1.70 | 1.44-2.00 | <0.001 |
| MI | 1.04 | 0.79-1.38 | 0.740 |
| PAD | 1.51 | 1.04-2.20 | 0.030 |
| DCM | 2.40 | 1.78-3.23 | <0.001 |
| Diabetes | 1.78 | 1.48-2.12 | <0.001 |
| Mitral valve disease | 1.64 | 1.38-1.95 | <0.001 |
| Aortic valve disease | 1.11 | 0.86-1.43 | 0.395 |
| CKD | 1.52 | 1.23-1.88 | <0.001 |
| Anemia | 0.83 | 0.66-1.04 | 0.106 |
| Malignancy | 0.95 | 0.66-1.39 | 0.824 |
| Prior stroke | 1.21 | 0.94-1.55 | 0.128 |
| Prior TIA | 1.03 | 0.66-1.60 | 0.877 |
| Age 65-74 | 1.24 | 1.03-1.50 | 0.023 |
| Age ≥75 | 1.01 | 0.83-1.21 | 0.973 |
| Age ≥80 years | 0.47 | 0.38-0.71 | <0.001 |
| Hypertension not well controlled | 1.48 | 1.22-1.80 | <0.001 |
| Liver disease | 0.76 | 0.45-1.26 | 0.297 |
| Bleeding events | 0.54 | 0.35-0.82 | 0.005 |
| AF was the main reason for the visit | 0.83 | 0.68-1.01 | 0.066 |
| Outpatient visit | 0.88 | 0.64-1.20 | 0.433 |
| Rate control | 1.37 | 1.05-1.78 | 0.020 |
| Rhythm control | 1.11 | 0.84-1.47 | 0.432 |

AF, atrial fibrillation; CAD, coronary artery disease; CI, confidence interval; CKD, chronic kidney disease; DCM, dilated cardiomyopathy; MI, myocardial infarction; OAC, oral anticoagulants; OR, odds ratio; PAD, peripheral artery disease; TIA, transient ischaemic attack.

Supplementary Table 6. Demographic, AF-related characteristics, physical finding, comorbidities, stroke and bleeding risk factors, baseline stroke and bleeding risk profile and AF management

| Variable | Patients with both newly-diagnosed AF and multimorbidity  n= 496 (21.9%) | Patients with both history of AF and multimorbidity n=1764 (78.1%) | p-value |
| --- | --- | --- | --- |
| Age, mean (SD), yrs | 69.7±10.2 | 70.5±9.8 | 0.103 |
| Age ≥75 yrs, n (%) | 192 (38.7) | 681 (38.6) | 0.966 |
| BMI, mean (SD), kg/m² | 27.6±4.6 | 28.0±4.4 | 0.169 |
| Male sex, n (%) | 260 (52.4) | 955 (54.1) | 0.191 |
| Current smoker, n (%) | 85 (17.1) | 176 (10.0) | <0.001 |
| Alcohol abuse, n (%) | 30 (6.0) | 61 (3.5) | 0.010 |
| Paroxysmal AF, n (%) | 314 (63.3) | 438 (24.8) | <0.001 |
| Persistent AF, n (%) | 0 (0.0) | 315 (17.9) | <0.001 |
| Permanent AF, n (%) | 0 (0.0) | 994 (56.3) | <0.001 |
| EHRA symptom score, mean (SD) | 2.5±0.8 | 2.1±0.8 | <0.001 |
| EHRA I, n (%) | 44 (8.9) | 364 (20.6) | <0.001 |
| EHRA II, n (%) | 219 (44.2) | 865 (49.0) | 0.053 |
| EHRA III, n (%) | 183 (36.9) | 460 (26.1) | <0.001 |
| EHRA IV, n (%) | 50 (10.1) | 74 (4.2) | <0.001 |
| Missing | 0 (0.0) | 1 (0.1) | 0.979 |
| NYHA class, mean (SD) | 1.7±0.9 | 1.9±1.1 | <0.001 |
| NYHA I, n (%) | 302 (60.9) | 882 (50.0) | <0.001 |
| NYHA II, n (%) | 80 (16.1) | 282 (16.0) | 0.939 |
| NYHA III, n (%) | 95 (19.2) | 428 (24.3) | 0.017 |
| NYHA IV, n (%) | 19 (3.8) | 172 (9.8) | <0.001 |
| SBP, mean (SD), mmHg | 137.7±24.1 | 135.1±22.1 | 0.021 |
| DBP, mean (SD), mmHg | 82.6±13.2 | 80.8±12.3 | 0.005 |
| Heart rate, mean (SD), beats per minute | 101.6±32.8 | 88.0±25.0 | <0.001 |
| Medical history, n (%) |  |  |  |
| Hypertension | 415 (83.7) | 1498 (84.9) | 0.495 |
| Hypertension well controlled^a^ | 249 (50.2) | 1027 (58.2) | 0.001 |
| Previous stroke/TIA | 55 (11.1) | 304 (17.2) | <0.001 |
| CAD | 191 (38.5) | 621 (35.2) | 0.178 |
| MI | 85 (17.1) | 278 (15.8) | 0.935 |
| Prior PCI/stenting | 66 (13.3) | 155 (8.8) | 0.009 |
| HF | 169 (34.1) | 976 (55.3) | <0.001 |
| Missing | 0 (0.0) | 1 (0.1) | 0.963 |
| LVEF < 40% | 88 (17.7) | 416 (23.6) | <0.001 |
| Diabetes mellitus | 150 (30.2) | 512 (29.0) | 0.581 |
| Prior bleeding | 14 (2.8) | 114 (6.5) | 0.002 |
| CKD | 77 (15.5) | 329 (18.7) | 0.109 |
| Aortic valve disease | 56 (11.3) | 243 (13.8) | 0.149 |
| Mitral valve disease | 140 (28.2) | 683 (38.7) | <0.001 |
| DCM | 25 (5.0) | 189 (10.7) | <0.001 |
| HCM | 9 (1.8) | 43 (2.4) | 0.414 |
| RCM | 1 (0.2) | 3 (0.2) | 0.883 |
| Hyperthyroidism | 24 (4.8) | 91 (5.2) | 0.298 |
| COPD | 70 (14.1) | 266 (15.1) | 0.601 |
| Sleep apnoea | 18 (3.6) | 34 (1.9) | 0.025 |
| PAD | 18 (3.6) | 102 (5.8) | 0.059 |
| Liver disease | 30 (6.0) | 64 (3.6) | 0.017 |
| Dementia | 19 (3.8) | 51 (2.9) | 0.285 |
| Hypercholesterolemia | 227 (45.8) | 762 (43.2) | 0.306 |
| Anaemia | 81 (16.3) | 287 (16.3) | 0.976 |
| Malignancy | 19 (3.8) | 99 (5.6) | 0.117 |
| Obesity | 111 (22.4) | 453 (25.7) | 0.125 |
| Number of comorbidities, mean (SD) | 4.2±1.9 | 4.9±2.2 | <0.001 |
| CHA_2_DS_2_-VASc, mean (SD) | 3.5±1.7 | 3.8±1.7 | 0.001 |
| CHA_2_DS_2_-VASc=0, n (%) | 3 (0.6) | 6 (0.3) | 0.408 |
| CHA_2_DS_2_-VASc=1, n (%) | 41 (8.3) | 127 (7.2) | 0.424 |
| CHA_2_DS_2_-VASc ≥ 2, n (%) | 452 (91.1) | 1631 (92.5) | 0.330 |
| HAS-BLED, mean (SD) | 1.8±1.0 | 2.2±1.2 | <0.001 |
| HAS-BLED ≥3, n (%) | 103 (20.8) | 687 (38.9) | <0.001 |
| *AF management settings, n (%)* |  |  |  |
| AF was the main reason for the hospitalization (at baseline visit) | 256 (51.6) | 750 (42.5) | <0.001 |
| Hospitalization due to ACS | 87 (17.5) | 113 (6.4) | <0.001 |
| Hospitalization due to HF | 71 (14.3) | 580 (32.9) | <0.001 |
| Hospitalization due to CCS | 17 (3.4) | 90 (5.1) | 0.121 |
| Hospitalization due to hypertension | 8 (1.6) | 47 (2.7) | 0.180 |
| Outpatient visit | 26 (5.2) | 146 (8.3) | 0.024 |
| Academic healthcare facility | 374 (75.4) | 1428 (81.0) | <0.001 |
| AF managed by a cardiologist | 389 (78.4) | 1378 (83.5) | 0.883 |
| AF managed by GP | 6 (1.2) | 42 (2.4) | 0.758 |
| AF managed by internal medicine specialist | 95 (19.2) | 264 (15.0) | 0.047 |
| AF managed by other specialist | 6 (1.2) | 80 (4.5) | 0.001 |
| *Stroke prevention (at baseline visit), n (%)* |  |  |  |
| No antithrombotic therapy | 39 (7.9) | 153 (8.7) | 0.581 |
| Overall OAC | 309 (62.3) | 1374 (77.9) | <0.001 |
| OAC alone | 235 (47.4) | 1151 (65.2) | <0.001 |
| Missing | 2 (0.4) | 1 (0.1) | 0.537 |
| VKA | 243 (49.0) | 1165 (66.0) | <0.001 |
| NOAC | 66 (13.3) | 209 (11.8) | 0.366 |
| SAPT alone | 96 (19.4) | 173 (9.8) | <0.001 |
| ASA (alone or with OAC) | 205 (41.3) | 399 (22.6) | <0.001 |
| DAPT alone | 50 (10.1) | 63 (3.6) | <0.001 |
| Dual antithrombotic therapy | 46 (9.3) | 170 (9.6) | 0.825 |
| Triple antithrombotic therapy | 28 (5.6) | 53 (3.0) | 0.005 |
| *Symptom management* |  |  |  |
| *Pharmacological AF therapies (at baseline visit), n (%)* |  |  |  |
| Rate control | 233 (47.0) | 1228 (69.6) | <0.001 |
| Missing | 12 (2.4) | 20 (1.1) | 0.318 |
| Rhythm control | 241 (48.6) | 444 (25.2) | <0.001 |
| Digoxin | 94 (19.0) | 525 (29.8) | <0.001 |
| Calcium channel blockers | 105 (21.2) | 391 (22.2) | 0.662 |
| Beta blockers | 360 (72.6) | 1323 (75.0) | 0.328 |
| Propafenone | 32 (6.5) | 129 (7.3) | 0.522 |
| Flecainide | 0 (0.0) | 1 (0.1) | 0.597 |
| Sotalol | 2 (0.4) | 15 (0.9) | 0.311 |
| Amiodarone | 177 (35.7) | 365 (20.7) | <0.001 |
| *Other therapy (at baseline), n (%)* |  |  |  |
| ACEi | 244 (49.2) | 863 (48.9) | 0.862 |
| AT1 receptor blockers | 93 (18.8) | 383 (21.7) | 0.163 |
| Loop diuretics | 187 (37.7) | 890 (50.5) | <0.001 |
| Statins | 237 (47.8) | 789 (44.7) | 0.204 |
| Number of drugs, mean (SD) | 4.8±1.6 | 5.0±1.5 | 0.026 |

ACS, acute coronary syndrome, AF, atrial fibrillation; ASA, acetylsalicylic acid; BMI, body mass index; CCS, chronic coronary syndrome, CAD, coronary artery disease, CHA_2_DS_2_-VASc, congestive heart failure, hypertension, age ≥ 75 years, diabetes, stroke/transient ischemic attack (TIA), vascular disease, age 65 to 74 years, sex category; CKD, chronic kidney disease; COPD, chronic obstructive pulmonary disease; DAPT, dual antiplatelet therapy; DBP, diastolic blood pressure; DCM, dilated cardiomyopathy; EHRA, European Heart Rhythm Association; GP, general practitioner, HAS-BLED: hypertension, abnormal renal/liver function, stroke, bleeding history or predisposition, labile International Normalised Ratio, elderly (age > 65 years), drugs or alcohol concomitantly; HCM, hypertrophic cardyomyopathy; HF, heart failure; LVEF, left ventricular ejection fraction; MI, myocardial infarction; NOAC, non-vitamin K oral anticoagulant; NYHA, New York Heart Association; OAC, oral anticoagulation; PAD, peripheral artery disease; PCI, percutaneous coronary intervention; SAPT, single antiplatelet therapy; SBP, systolic blood pressure; SD, standard deviation; TIA, transient ischaemic attack; VKA, vitamin K antagonists.

Supplementary Table 7. Independent predictors of OAC use in multimorbid patients and newly-diagnosed AF

|  | **Univariate analysis** | | | **Multivariate analysis** | | |
| --- | --- | --- | --- | --- | --- | --- |
| **Variable** | **OR** | **95% Confidence interval** | **p-value** | **OR** | **95% Confidence interval** | **p-value** |
| Mean age, years | 0.98 | 0.96-0.99 | 0.045 | 0.98 | 0.95-0.99 | 0.018 |
| Male sex | 1.13 | 0.79-1.61 | 0.492 |  |  |  |
| Capital city | 1.44 | 1.10-2.05 | 0.049 | 1.59 | 1.03-2.46 | 0.035 |
| Non-emergency centre | 0.37 | 0.23-0.59 | <0.001 |  |  |  |
| University centre | 1.39 | 0.88-2.18 | 0.160 |  |  |  |
| Cardiologist | 1.32 | 0.85-2.02 | 0.214 |  |  |  |
| Alcohol abuse | 1.12 | 0.53-2.34 | 0.767 |  |  |  |
| Paroxysmal AF | 0.28 | 0.19-0.41 | <0.001 | 0.29 | 0.19-0.46 | <0.001 |
| Obesity | 0.89 | 0.58-1.37 | 0.598 |  |  |  |
| Hypertension | 2.01 | 1.22-3.32 | 0.006 |  |  |  |
| Heart failure | 1.33 | 0.92-1.93 | 0.133 |  |  |  |
| CAD | 0.16 | 0.11-0.25 | <0.001 | 0.12 | 0.11-0.28 | <0.001 |
| MI | 0.48 | 0.23-0.99 | 0.048 | 0.30 | 0.13-0.72 | 0.007 |
| PAD | 1.11 | 0.43-2.85 | 0.827 |  |  |  |
| DCM | 4.76 | 1.76-2.91 | 0.002 | 4.53 | 1.51-5.19 | 0.002 |
| Diabetes | 0.55 | 0.37-0.82 | 0.003 |  |  |  |
| Mitral valve disease | 1.95 | 1.31-2.91 | 0.001 |  |  |  |
| Aortic valve disease | 1.44 | 0.82-2.51 | 0.206 |  |  |  |
| CKD | 1.03 | 0.64-1.68 | 0.898 |  |  |  |
| Anemia | 0.68 | 0.42-1.10 | 0.118 |  |  |  |
| Malignancy | 0.80 | 0.32-2.02 | 0.633 |  |  |  |
| Prior stroke | 1.06 | 0.56-2.01 | 0.861 |  |  |  |
| Prior TIA | 0.83 | 0.28-2.42 | 0.732 |  |  |  |
| Age 65-74 | 0.99 | 0.67-1.46 | 0.973 |  |  |  |
| Age≥75 | 0.84 | 0.59-1.21 | 0.359 |  |  |  |
| Hypertension not well controlled | 1.08 | 0.73-1.60 | 0.717 |  |  |  |
| Liver disease | 1.48 | 0.70-3.12 | 0.301 |  |  |  |
| Bleeding events | 0.43 | 0.13-1.40 | 0.162 |  |  |  |
| AF was the main reason for the visit | 2.25 | 1.57-3.23 | <0.001 | 2.69 | 1.82-3.97 | <0.001 |
| Outpatient visit | 1.31 | 0.59-2.90 | 0.499 |  |  |  |
| Rate control | 2.15 | 1.50-3.10 | <0.001 | 2.66 | 1.81-3.92 | <0.001 |
| Rhythm control | 0.70 | 0.49-0.99 | 0.044 |  |  |  |

AF, atrial fibrillation; CAD, coronary artery disease; CKD, chronic kidney disease; DCM, dilated cardiomyopathy; MI, myocardial infarction; OAC, oral anticoagulants; OR, odds ratio; PAD, peripheral artery disease; TIA, transient ischaemic attack.
